# What are the Key Characteristics of a ‘Good’ Psychotherapy? Calling for Ethical Patient Involvement

**DOI:** 10.3389/fpsyt.2020.00406

**Published:** 2020-06-02

**Authors:** Heike Gerger, Antje Frey Nascimento, Cosima Locher, Jens Gaab, Manuel Trachsel

**Affiliations:** ^1^Division of Clinical Psychology and Psychotherapy, Faculty of Psychology, University of Basel, Basel, Switzerland; ^2^Department of General Practice, Erasmus MC University Medical Center, Rotterdam, Netherlands; ^3^School of Psychology, University of Plymouth, Plymouth, United Kingdom; ^4^Department of Anesthesiology, Critical Care and Pain Medicine, Boston Children's Hospital, Harvard Medical School, Boston, MA, United States; ^5^Faculty of Medicine, Institute of Biomedical Ethics and History of Medicine, University of Zurich, Zurich, Switzerland; ^6^Clinical Ethics Unit, University Hospital of Basel, Basel, Switzerland

**Keywords:** psychotherapy, patient-centered care, empirically supported treatment, evidence-based practice, patient autonomy

## Abstract

**Objective:**

The evidence-based practice movement clearly defines the relevant components of a good treatment. In the present article, we elaborate on how the active involvement of patients within psychotherapy can and should be increased in order to respect ethical considerations. Our arguments complement the requirements of evidence-based practice, and are independent of the actual psychotherapeutic treatment approach being used.

**Method:**

Theoretical and ethical analysis.

**Results:**

In order to respect patient autonomy, psychotherapy needs to be transparent and honest when it comes to disclosing the relevant factors for promoting therapeutic change. It has been argued that ethical informed consent needs to include empirically supported patient information. In this paper we go one step further: we outline that fully respecting ethical considerations in psychotherapeutic treatment necessarily calls for acknowledging and strengthening the active role of patients in the course of psychotherapy. Accordingly, patients need not only to be informed openly and transparently about the planned treatment, the treatment rationale, and the expected prognosis of improvement in the course of psychotherapy, but they also need to be actively involved in the decision-making process and during the entire process of psychotherapeutic treatment.

**Conclusions:**

Our arguments support the tendency that can be observed in health care in recent years towards more active patient involvement across different health-care domains, but also in clinical research. This article offers an ethical perspective on the question what defines a ‘good psychotherapy', which, hopefully, will help to leave behind some of the ongoing psychotherapy debates and move the field forward.

## Introduction

Since Eysenck's provocative conclusion in 1952 that psychotherapy doesn't facilitate recovery from mental disorders (1), it has been a major goal of psychotherapy research to prove the *efficacy* and *effectiveness* of psychotherapy. With the adoption of the criteria of evidence-based medicine (2–4) to psychotherapy outcome research, the proof of efficacy became necessary for a psychotherapeutic treatment to be considered empirically supported and thus to be recommended for clinical practice (5, 6). Within the evidence-based practice framework, however, a broad perspective is taken into account acknowledging that beyond the theory-driven ingredients of the intervention, research evidence points to relevant characteristics of the patient or client, as well as of the treatment provider, and the interactive process of treatment as relevant aspects (e.g., 7, 8). Following previous claims regarding the patient as being a, if not the most important factor contributing to psychotherapy effects (9–11), the present paper will focus on the role of patients within the course of psychotherapeutic treatment.

Calls for more active patient involvement in psychotherapy are not new, and have their origins within humanistic and positive psychology, focusing on each person's potential for growth (12). For instance, Rogers who developed the person-centered psychotherapy approach, stated in 1963 “we could say that in the optimum of therapy the person rightfully experiences the most complete and absolute freedom” (13, p.25). In 1994, Bergin and Garfield wrote that “clients are not inert objects upon which techniques are administered…”, and further, “as therapists have depended more upon the client's resources, more change seems to occur” (14, p.825–826, as cited in 15 p.84). In addition, literature on resilience points out the potential of client- and patient-associated factors to be related with self-directed change and self-healing, and to contribute to health-improvements (16, 17). Interestingly, Maslow's theory of human motivation (18) seems to have anticipated these developments by highlighting the importance of self-fulfillment and self-actualization as forming the basis for personal functioning and resilience. As a practical example of how the patient can be given the lead in psychotherapy, a patient-led approach has been suggested and evaluated, which gives the patient responsibility for the planning and structuring of psychotherapy (19, 20).

Our paper will build on previous literature pointing out the relevance of patient or client involvement in psychotherapy. We will complement this line of research by adding an ethical perspective and deducing that if transferring ethical considerations to psychotherapy practice, actively involving patients in the entire psychotherapeutic process is not only possible but also necessary from an ethical point of view, although doing so, might be a challenge in certain cases. From a practical point of view, providing a clear and evidence-based guideline on how to realize the goal of ethical patient involvement within practice is beyond the scope of this article which rather aims at raising awareness on the relevance of ethical considerations in psychotherapy. However, in some instances we will provide examples on how the suggested goal of ethical patient involvement may be translated or has been translated to clinical practice.

## Ethics in Psychotherapy

In medical ethics, the applied ethics approach of *principlism* forms the basis for many ethical guidelines, and postulates four ethical principles (21): first, *respect for autonomy* (self-determination); second, *beneficence* (do good); third, *non-maleficence* (do no harm); and fourth, *justice* (social distribution of benefits and burdens). In dealing with ethical questions, conflicts, and/or dilemmas, each of the four principles needs to be specified and balanced, recognizing that there is no hierarchical order of the four principles from the outset (21).

Not only in medicine in general but also in psychotherapy, the applied ethics approach of principlism may be an attractive framework for moral decision-making because it is undogmatic, open with regard to any theory of normative ethics, liberal, transparent, and rational. In recent years, an increased emphasis on ethics in psychotherapy can be observed, which may complement the available ethical codes of conduct in several countries (e.g., 22, 23). Over the last five years, the publication of textbooks for practitioners (e.g., 24, 25), of scientific journal articles (e.g., 26, 27), of article collections and special issues (e.g., 28, 29), and the publication of the “Oxford Handbook of Psychotherapy Ethics” (30) reflect the increase in interest and knowledge in this field.

In this context, ethical arguments have recently been raised to call for adequate patient information in psychotherapy, based on empirical evidence. Ethical patient information is required to provide all the information that is necessary for a patient to make an informed decision concerning a suggested treatment plan (6, 31–33). In a recent systematic review, Lamont-Mills and colleagues summarized the evidence on the role of confidentiality and informed consent in counselling and psychotherapy (34). They concluded that within clinical practice, psychotherapists apply standardized informed consent templates but they also state that we know only little about the actual adequacy of informed consent obtainment in psychotherapy as well as on the patients' own understanding of informed consent and confidentiality. We will argue in the present article that despite the necessity to simply inform patients about the suggested treatment, for instance by using standardized informed consent templates, the patients should be actively involved in the entire therapeutic process in an individualized way. We will elaborate on how this can be done and which aspects are to be considered when doing so.

## Ethical Patient Involvement

### Providing an Individualized, Plausible, Comprehensive, and Honest Treatment Rationale

From an ethical point of view, transparency in the conduct of psychotherapy is essential and serves to respect and protect patients' autonomy (31–33). To this effect, treatment rationales need to be plausible and clear, but also compelling (35). It is indispensable, however, that honesty is warranted, and that exaggerations are avoided (26). Therefore, treatment rationales need to be based on evidence-based and empirically supported research findings (5, 31, 36, 37). Moreover, the therapist's language must be adapted with respect to the patient's own language (38), and the patient's individual context needs to be considered. The provision of individualized honest and transparent treatment rationales is key for assuring patient autonomy in that they enable a patient to decide for or against initiating psychotherapeutic treatment in an informed manner (32, 33). In turn, a higher credibility of an initiated treatment, as perceived by patients at an early stage of treatment, as well as patients' outcome expectations have been shown to be significantly associated with treatment outcomes (39, 40). Yet, it is important to note that patient information does not need to include the explanation of complex psychological theories if not warranted. Research on the open and transparent administration of placebo treatment for instance has demonstrated benefits of the provision of rather short, yet compelling treatment rationales even in the absence of an active treatment (41–43). However, the integration of patient's individual views and perspectives within the framework of evidence-based treatment might seem as a contradiction. But recent research proposes to allow for more variability and evidence-based therapist flexibility, for instance, within the context-responsive psychotherapy integration framework. The application of this framework in clinical practice realizes personalization of psychotherapy by therapists' responsiveness to patients' characteristic, for instance their treatment-related beliefs (44). In summary we argue that the process of informing a patient regarding a treatment rationale within psychotherapy should actively consider the patient’s own perspectives in order to respect the patient’s autonomy.

### Defining the Outcome of Psychotherapeutic Treatment Including the Patient Perspective

To take the ethical principles of respect for autonomy and beneficence seriously, patients should also be included in an active manner in the process of defining the domain of outcome of psychotherapeutic treatment. Strupp, Fox (45) were among the first ones to highlight the relevance of the patients' own perspective in outcome assessment in psychotherapy. Besides the core symptoms as defined in diagnostic manuals (e.g. the Diagnostic and Statistical Manual of Mental Disorders DSM-V (46) or the International Statistical Classification of Diseases ICD-10 (47), a range of additional health-related outcomes exist which might be considered relevant as well (e.g., 48, 49). Alternative treatment outcomes might encompass such aspects as quality of life, well-being, self-efficacy, and social relationships, among many more. For instance, meaning of life has been described as being correlated with psychopathological symptoms (50, 51), and as a possible alternative target outcome of psychotherapy (52). In practice, the aim to include patient's perspectives can be realized in diverse ways, including for instance the use of multidimensional routine treatment outcome packages (53), the assessment of personal target complaints (54), but may also include the use of the “miracle question” or of “scaling questions” (55, 56). It might be argued, that for many patients, particularly those who are ambivalent about change, discussions about treatment goals and desired outcomes of treatments might be challenging if not impossible. De Shazer and Isebaert give an informative overview on how respect for patient autonomy can be realized within inpatient and outpatient psychotherapeutic treatment of alcohol abuse, which has traditionally been described as being difficult to treat, by focusing on “what patients want from therapy” (57, p.51). Thus, in order to meet the ethical principles of respect for patient autonomy and beneficence, the identification of the most relevant health dimension to be improved in the course of a psychotherapeutic treatment needs to actively include and respect the patient's own perspective.

### Discussing the Evidence Openly With the Patient

In general, psychotherapy is required to have beneficial effects at least on the core symptoms of a certain diagnosis in order to be considered evidence-based. Unfortunately, due to the scarcity of research on other outcome domains than symptom improvement, we know only little about psychotherapy effects on other outcome domains so-far. In order to respect the ethical principle of respect for the patient autonomy, these lacks of evidence should be discussed with patients. Further, the available evidence on potentially occurring unwanted effects or side effects in the course of psychotherapy is very limited (6, 58). Nevertheless, in accordance with the ethical principles of autonomy and of non-maleficence, the potential risks as well as lacks of available evidence needs to be disclosed to patients openly in order to allow for the patients to make an informed decision.

Likewise, in accordance with the ethical principles of beneficence and of non-maleficence, the influence of therapists, proven to contribute significantly to treatment effects (59), should be taken into account while discussing potential treatment outcomes (60). Research has shown that not all therapists are similarly effective (61–63). Yet, patients wish to obtain information on their therapist's performance level (64). Therefore, it is very important to discuss with a patient the possibility that a mismatch between the patient and the therapist may limit potentially beneficial treatment effects, in order to avoid the patient to conclude that an unsuccessful course of treatment was his or her own fault. Within the therapeutic process, therapists could raise this issue occasionally, and offer the patient the possibility to swap therapists, or change treatment.

### Discussing the Potential Course of Symptom Improvement

Several meta-analyses have shown that short-term effects of psychotherapeutic treatments may differ considerably from long-term effects (65–68). From a clinical as well as from an ethical and financial perspective, one could argue that a treatment would need to contribute to long-lasting, sustainable benefits in order to be recommended for clinical practice (69, 70). However, from a patient's perspective, even short-term improvements may be considered relevant, and may considerably impact well-being. For instance, in the context of medically unexplained symptoms, it has been argued that given the high personal burden associated with the mostly chronic course of symptoms without the hope for complete recovery, even short-term symptom relief might be considered as highly relevant by individual patients (67). In addition, therapists might argue that short-term deteriorations of symptoms or well-being may be part of the therapeutic process, which eventually lead to long-term improvements. For instance, crying during treatment sessions has been described as relieving distress and arousal, but can also be perceived as stressful in itself, and as contributing to the experience of increasing arousal (71). In this context, however, the ethical principle of doing no harm (nonmaleficence), for example the danger of introducing negative expectations, and increasing the risk for the occurrence of nocebo effects needs some attention (72). It could further be argued, that talking about potentially occurring symptom deteriorations might decrease patients' commitment to treatment and the therapeutic relationship. Previous research, however, identified potential and diverse ways how to deal with such difficult situations within psychotherapy (57, 73, 74). It is important however to respect patients' autonomy by allowing the patients an informed decision whether or not to adhere to a suggested treatment plan. Accordingly, the discussion of the potential course of symptoms over time, which may include temporal symptom deteriorations or possible discrepancies between expected short- and long-term effects of psychotherapy, requires a particularly sensitive and individualized process, which necessarily takes into consideration a patient's previous experience, expectations, and other patient-related characteristics.

### Considering Patients’ Previous Experiences

In the course of ethically sound psychotherapy, exploring and discussing patients' previous treatment experiences as well as their subjective illness narratives (i.e. their own understanding regarding how a certain illness is perceived, understood and managed; 75) seems most relevant. Besides exploring patients' previous treatment experiences, it is also relevant to explore what patients themselves have been doing in the past in order to deal with difficulties and crises in their lives, as well as pointing out previous successes and achievements in handling previous problems (56). Such explorations may give important hints regarding patients' strengths and resources, and can contribute to creating awareness and positive expectations while strengthening the patients' own capacity to cope with problems. In this sense, psychotherapy can be described as contributing to transforming non-adaptive narratives into adaptive ones (76). Thus, “psychotherapy is not simply the vehicle for the delivery of psychological ingredients but is, rather, a highly entwined system that uses language to construct or, better said, reconstruct the patient's interpretation of the world.” (77, p. 862). In practice, acknowledging patients previous experiences can be done for instance by responding individually to patients unique characteristics and emerging scenarios (44), or by the exploration of so-called exceptions of the problem (57).

### Monitoring Treatment Progress

In shifting the focus towards the patient's perspective in clinical research, patient-reported outcomes measures (PROMs) were originally applied in clinical research in order to quantitatively assess health outcomes from the patient's perspective (78, 79). Meanwhile, however, they are increasingly used in clinical practice to monitor and improve health care for individual patients (80). Also in the specific context of psychotherapy feedback systems have been introduced (81, 82), which can be used to inform the therapist about the actual course of a particular patient, and may facilitate personalized planning and adapting of processes within psychotherapy. For example, the application of the routine outcome monitoring has been shown to be superior to “clinical judgment in predicting patients who are on or off track for treatment success” (82, p.459). Electronic feedback systems just as the use of PROMs in clinical practice are assumed to help improving the communication between patients and clinicians, to foster a shared decision-making process, and to develop and monitor personalized care plans (83). However, they are not to replace the necessary exchange between a clinical psychologist and the patient regarding the patient's idiosyncratic perceptions of and attitudes towards the course of treatment.

## Conclusions

During the last decades, psychotherapy research just as other areas of mental health care research have largely focused on the one hand on the identification of clear-cut definitions of mental disorders with several revisions of the defining criteria over the years (84–86), and on the other hand on the identification of treatments that are specifically tailored to a diagnosis and which were assumed to help eliminate the defining symptoms of a diagnosis better than other more generic treatments (49, 87). This dominating view made psychotherapy research endeavors initially focus on proving the *efficacy* of psychotherapeutic treatments. Over the years, the focus slightly moved towards more naturalistic investigations of the *effectiveness* of treatments, and in recent years, the *efficiency* of psychotherapy gained more research interest, and an increase in publications on the cost-effectiveness of psychotherapeutic treatments can be observed (see e.g., 88–92).

In addition to *efficacy*, *effectiveness* and *efficiency*, however, *ethical considerations* are most relevant when talking about criteria of a ‘good' psychotherapy. We have argued that treatment recommendations, which respect the ethical principles of respect for autonomy, beneficence, non-maleficence, and justice, reflect an individualized *patient-centered process* that should incorporate the relative importance of individual patient's history, values and needs.

### Patients as Partners

We showed that an active involvement of patients is most relevant when including ethical principles in psychotherapy decision-making and practice. In this understanding, based on the ethical principles of respect for autonomy and beneficence, patients are to be seen as partners in clinical practice. In addition, it has been argued recently that patients' perspectives should be included in clinical research as well, for instance in study design and governance (93, 94), in order to increase the relevance of research findings for the patients and the actual clinical practice outside of the academic setting. These claims are nicely summarized in The BMJ's patient partnership strategy (95), as well as in the statements published by the Patient-Centered Outcomes Research Institute in the US (96, 97), as well as the National Institute for Health Research in the UK (98).

The addition of ethical considerations to the debate strengthens previous calls for shifting the focus from the treatment itself towards other relevant aspects of psychotherapy (99, 100). In particular, patients themselves as active agents within psychotherapy need more attention, including their idiosyncratic experiences with psychotherapy, as well as their perspectives on health and illness (i.e. their illness and health narratives), their moral and normative values, but also their financial and time-wise investments when initiating psychotherapy (10, 37, 99, 101).

Our call for more patient involvement in the course of psychotherapy is not new. In fact, some psychotherapeutic approaches exist which are not based on theoretical assumptions about the etiology of mental problems or disorders, but which focus more on the idiosyncratic process within psychotherapy. For instance, in solution focused brief therapy the patient is seen as the expert of therapeutic change rather than the therapist (102). Likewise, humanistic approaches, such as person-centered psychotherapy in general and, in particular, motivational interviewing, rely on establishing and safeguarding of a therapeutic alliance to allow and foster change. They have a strong focus on the processes of change rather than on etiological models or the adherences to protocols and manuals (103, 104). The three outlined psychotherapeutic approaches have in common that patients' views, experiences, values, and needs are actively involved throughout the whole course of treatment—an expression of the ethical principle of respect for autonomy, while the therapist supports and guides rather than directs the therapeutic process.

### One Size Does Not Fit All

We conclude that the ethical principles of patient autonomy, beneficence, non-maleficence and justice can best be respected within an individualized and patient-centered process within psychotherapeutic treatment. In this context the principles described in person-centered psychotherapy (104) seem to be of high relevance just as the processes described for instance in the context of motivational interviewing (103), or in the therapist's attitude of ‘not-knowing' in solution focused brief therapy (56, 102).

We have argued that throughout the course of psychotherapy, therapists need to remain in exchange with the patient regarding the process of change during the course or after finishing psychotherapy. This exchange may include discussions about first, multiple dimensions of potential treatment outcomes (i.e. not only focusing on symptom improvement but on a broader range of health-related outcomes), second potential symptom worsening or otherwise occurring adverse events, third, the long-term perspectives of expected treatment effects, and fourth, the costs of a psychotherapeutic treatment, financially but also time-wise. All four of them may differ considerably between individuals with respect to their actual content and the relevance of one aspect compared to the others. This exchange between patient and therapist should be tailored to individual patients and should be guided by their previous experiences, their individual illness narratives, their values, and needs.

It is important to keep in mind, however, that comprehensive patient information also bears potential risks. Just as in medical treatment, where unwanted events can be elicited by emphasizing them (e.g., 105, 106), the occurrence of so-called nocebo effects has also been discussed in the context of psychotherapy (72, 107–109). From an ethical perspective, risks should neither be exaggerated nor be concealed by a practitioner (110). In order to respect ethical principles, and in order to avoid the occurrence of nocebo-effects (maleficence), in the case of doubt, patients should explicitly be asked whether they care for knowing all details regarding potential risks that may be associated with initiating psychotherapeutic treatment thereby meeting the ethical principle of respect for the patient's autonomy.

Following our arguments there is probably not one recommendable ‘good' or ‘best' psychotherapy. Rather, the evaluation of certain psychotherapeutic treatments as a ‘good' psychotherapy for a certain patient always constitutes an individual decision (99), and may thus differ across individuals depending for instance on their backgrounds, clinical conditions, personal values, and their illness and health narratives. The addition of the *ethical perspective* to the evaluation of psychotherapeutic treatments may therefore be seen as a key element which shifts the focus from a treatment itself (i.e. its *efficacy*, *effectiveness* and *efficiency*) towards the patient, and thus necessarily strengthens the patient's active role within psychotherapy.

## Author Contributions

HG, AN, CL, JG, and MT wrote and reviewed the manuscript.

## Funding

CL received funding for this project from the Swiss National Science Foundation (SNSF): P400PS_180730.

## Conflict of Interest

The authors declare that the research was conducted in the absence of any commercial or financial relationships that could be construed as a potential conflict of interest.
